# Unraveling the intricate link: gut microbiota and recurrent spontaneous abortion

**DOI:** 10.3389/frph.2026.1801173

**Published:** 2026-05-21

**Authors:** Dan Li, Xiaoqiang Liu, Zhaogang Teng, Chenxi Yang, Xiaoxia Chen, Ping Tan

**Affiliations:** 1 Department of Nephrology, Qingdao Central Hospital, University of Health and Rehabilitation Sciences, Qingdao, China; 2 Reproductive Medicine Center, Qingdao Women and Children’s Hospital, Qingdao, China; 3 Department of Obstetrics and Gynecology, Xihe County People’s Hospital, Longnan, China; 4 Department of Obstetrics and Gynecology, Affiliated Hospital of Qingdao University, Qingdao, China

**Keywords:** gut microbiota, immune regulation, inflammation, metabolites, recurrent spontaneous abortion

## Abstract

Recurrent spontaneous abortion (RSA) affects 1%–5% of reproductive-age couples, and nearly 50% of cases remain unexplained. This review summarizes recent evidence to elucidate the association between gut microbiota dysbiosis and RSA. We confirm that RSA patients exhibit decreased alpha diversity, reduced levels of *Bacteroidetes*, *Lactobacillus*, and *Bifidobacterium*, and elevated *Proteobacteria* and *Escherichia-Shigella*. Gut dysbiosis contributes to RSA through three core pathways: disrupted T helper 17/regulatory T cell balance leading to maternal–fetal immune intolerance; insufficient short-chain fatty acid production and elevated trimethylamine N-oxide causing metabolic disorders; and disrupted estrogen and progesterone metabolism via microbial hydroxysteroid dehydrogenases triggering endocrine dysfunction. We also clarify the bidirectional causal relationship between gut dysbiosis and RSA, summarize inconsistent findings across studies, and highlight the potential of probiotics and dietary interventions. This review provides mechanistic insights and clinical implications for microbiota-targeted strategies in unexplained RSA.

## 1 Introduction

### 1.1 Background

Recurrent spontaneous abortion (RSA) is a distressing and complex medical condition that significantly impacts the physical and mental health of women. It is estimated that approximately 1% to 5% of couples of reproductive age experience RSA, with the risk increasing with advanced maternal age and the number of previous miscarriages (1–3). RSA not only causes physical harm, such as uterine damage and increased risk of infection, but also leads to severe psychological stress, including depression, anxiety, and infertility-related distress (4).

The causes of RSA are multifactorial and include genetic, anatomical, endocrine, immunological, thrombophilic, and environmental factors. However, despite extensive research, the etiology remains unknown in about 50% of cases, known as unexplained RSA (URSA) (5). In recent years, emerging evidence has suggested a potential link between the gut microbiota and RSA, highlighting the gut microbiota-host axis as a novel area of research in reproductive medicine (6, 7).

The gut microbiota, a complex community of microorganisms residing in the gastrointestinal tract, plays a crucial role in maintaining human health. It is involved in various physiological processes, such as digestion, nutrient absorption, metabolism, and immune regulation. Dysbiosis of the gut microbiota, characterized by alterations in the composition and function of the microbial community, has been associated with a wide range of diseases, including inflammatory bowel disease, obesity, diabetes, and mental health disorder (8). Given the close connection between the gut microbiota and the immune system, as well as the importance of immune regulation in pregnancy, understanding the relationship between the gut microbiota and RSA may provide new insights into the pathogenesis and management of this condition.

### 1.2 Definition of RSA

RSA is defined as the occurrence of two or more consecutive spontaneous abortions before 28 weeks of gestation (9), and some studies may include biochemical pregnancies or consider three or more miscarriages as the diagnostic criterion. The incidence of RSA varies among different populations, with reports ranging from 1% to 5% (1–3).

RSA can have a profound impact on the psychological well-being of affected women. The repeated loss of pregnancies often leads to feelings of grief, guilt, and hopelessness, which can significantly reduce their quality of life. In addition to the emotional toll, RSA also poses a physical risk, as multiple abortions can increase the risk of uterine perforation, adhesions, and infections, potentially affecting future fertility and pregnancy outcomes (5).

### 1.3 The gut microbiota: an overview

The gut microbiota is a vast and diverse community of microorganisms, including bacteria, archaea, fungi, and viruses, that inhabit the human gastrointestinal tract. It is estimated that the gut microbiota contains over 100 trillion microorganisms, representing thousands of different species, with a combined gene repertoire (the microbiome) that is approximately 100-fold larger than the human genome (10). The composition of the gut microbiota is highly individualized and influenced by various factors, such as genetics, diet, lifestyle, environment, medications (especially antibiotics), and early-life events (e.g., mode of delivery and breastfeeding) (11). The major phyla of bacteria in the human gut are Firmicutes, Bacteroidetes, Actinobacteria, Proteobacteria, and Verrucomicrobia, with Firmicutes and Bacteroidetes typically accounting for the majority of the bacterial population (12).

The gut microbiota performs numerous essential functions in the human body. It aids in the digestion and absorption of nutrients, such as complex carbohydrates, proteins, and fats, by producing enzymes that the human body cannot synthesize. For example, certain bacteria in the gut can break down dietary fiber into short-chain fatty acids (SCFAs), which are not only an important energy source for the colonocytes but also have systemic effects on metabolism, inflammation, and immune function (13). The gut microbiota also plays a crucial role in the synthesis of vitamins, such as vitamin K and B-vitamins, and the production of neurotransmitters, including serotonin, which is involved in mood regulation (14). Furthermore, the gut microbiota is a key component of the immune system. It helps to educate and modulate the immune response, promoting immune tolerance to harmless antigens while maintaining the ability to mount an effective defense against pathogens. A balanced gut microbiota can prevent the over-activation of the immune system and reduce the risk of inflammatory and autoimmune diseases (15). It does this by interacting with immune cells in the gut-associated lymphoid tissue (GALT), such as T cells, B cells, and dendritic cells, and influencing the production of cytokines and other immune mediators (16). In summary, the gut microbiota is an integral part of the human body, with far-reaching effects on health and disease. Any disruption to the normal balance of the gut microbiota, known as dysbiosis, can have significant consequences for the host's physiological and immunological functions, potentially leading to the development of various diseases, including those related to reproduction such as RSA.

Despite advances in reproductive medicine, the pathogenesis of unexplained RSA remains unclear. Emerging evidence suggests that the gut microbiota plays a critical role in pregnancy maintenance and immune tolerance. However, the causal direction, consistent microbial markers, and actionable mechanisms linking gut dysbiosis to RSA have not been systematically summarized. This review aims to integrate the latest clinical and mechanistic studies, clarify the bidirectional relationship between gut dysbiosis and RSA, summarize consistent and inconsistent findings, and evaluate potential interventions. By doing so, we hope to provide a theoretical foundation for early risk assessment, mechanistic research, and microbiome-targeted therapy for RSA.

## 2 Methodology of research

### 2.1 Literature search strategy

A comprehensive literature search was conducted to identify relevant studies on the relationship between gut microbiota and RSA. The following databases were searched: PubMed, Embase, Web of Science, and the Cochrane Library. These databases were chosen for their comprehensive coverage of medical and scientific literature, ensuring a wide-ranging search.

The search terms included combinations of the following keywords: “gut microbiota”, “intestinal microbiota”, “recurrent spontaneous abortion”, “recurrent miscarriage”, “pregnancy loss”, “dysbiosis”, “microbiome”, and related terms. Boolean operators (AND, OR) were used to combine the search terms to ensure a focused and comprehensive search. For example, “(gut microbiota OR intestinal microbiota) AND (recurrent spontaneous abortion OR recurrent miscarriage)”.

The search was limited to articles published in the English language in the past five years to ensure the inclusion of the most up-to-date research. Additionally, the search was restricted to human studies to directly address the relationship in the context of human reproduction.

After the initial search, the titles and abstracts of the identified articles were screened by two independent reviewers. Articles that clearly did not meet the inclusion criteria, such as those not related to the gut microbiota and RSA, were excluded. Full-text articles of the remaining potentially relevant studies were then retrieved and carefully evaluated. During this stage, studies were further excluded if they did not provide original data on the gut microbiota in relation to RSA, were review articles without new data, or were case reports with insufficient data for analysis.

### 2.2 Selection criteria for high-quality studies

High-quality studies were prioritized to ensure the reliability and validity of the evidence presented in this review. The following types of studies were given preference:

**1. Randomized Controlled Trials (RCTs)**: RCTs are considered the gold standard in clinical research as they involve random allocation of participants to intervention and control groups, minimizing bias. In the context of gut microbiota and RSA, RCTs could involve interventions such as probiotic supplementation to modulate the gut microbiota and then observe the impact on RSA rates. For example, a well-designed RCT might randomly assign women with a history of RSA to either a probiotic-treated group or a placebo-treated group and follow them through subsequent pregnancies to compare the incidence of miscarriage (17).
**2. Cohort Studies**: Cohort studies, especially prospective cohort studies, can provide valuable information on the natural history of the relationship between gut microbiota and RSA. These studies follow a group of individuals over time, allowing for the assessment of the temporal relationship between changes in the gut microbiota and the occurrence of RSA. For instance, a large-scale prospective cohort study could enroll women of reproductive age, analyze their gut microbiota profiles at baseline, and then track their pregnancy outcomes over several years to determine if specific gut microbiota features are associated with an increased risk of RSA (18).
**3. Systematic Reviews and Meta-Analyses**: These studies synthesize the results of multiple primary studies, providing a more comprehensive and objective overview of the evidence. A high-quality systematic review or meta-analysis on the gut microbiota and RSA would critically appraise and combine the data from relevant RCTs, cohort studies, and other eligible studies. This can help to identify common trends, quantify the strength of the association, and assess the overall quality of the evidence. For example, a meta-analysis might pool the results of several RCTs on probiotic use in preventing RSA to determine the overall effectiveness of this intervention (19).

When evaluating the quality of studies, several factors were considered. These included the study design (e.g., adequacy of randomization, blinding, and control groups in RCTs), sample size (larger sample sizes generally provide more reliable results), data collection methods (e.g., accurate and standardized measurement of gut microbiota composition and pregnancy outcomes), and the presence of potential biases (such as selection bias, confounding factors, and reporting bias). Only studies that met strict quality criteria were included in the review to ensure that the conclusions drawn were based on the most reliable evidence available.

### 2.3 Literature screening and quality evaluation results

A total of 186 articles were initially retrieved from the four databases. After duplicate removal, 127 articles remained. After screening titles and abstracts, 92 irrelevant articles were excluded, and 35 full-text articles were assessed for eligibility. Finally, 19 articles met the inclusion criteria and were included in this review. The detailed screening process and quality evaluation are shown in Table 1.

**Table 1:** Literature screening flow and quality evaluation.

Screening step	Number of articles	Exclusion reason
Initial retrieval	186	-
Duplicate removal	59	Duplicate publications
Title & abstract screening	92	Irrelevant topics; non-human studies; non-English; not about gut microbiota and RSA
Full-text evaluation	16	No original data; small sample size, low methodological quality
Final included	19	-

The quality of the 19 included studies was evaluated using predefined criteria: (1) Randomized controlled trials (RCTs, *n* = 4): Adequate randomization, double-blinding, complete follow-up, and clear outcome definitions. (2) Prospective cohort studies (*n* = 5): Clear baseline gut microbiota detection, long-term follow-up, and standardized pregnancy outcome assessment. (3) Case-control studies (*n* = 8): Well-matched groups, standardized 16S rRNA sequencing, and adjustment for confounders. (4) Systematic reviews & meta-analyses (*n* = 2): Clear inclusion/exclusion criteria, low heterogeneity, and rigorous statistical methods.

All included studies were confirmed to be high-quality evidence focused on gut microbiota and RSA.

## 3 Gut microbiota and pregnancy

### 3.1 Normal changes in gut microbiota during pregnancy

During pregnancy, the gut microbiota undergoes significant alterations in composition, abundance, and function, which are thought to be an adaptive response to support the physiological changes associated with pregnancy (20).

#### 3.1.1 Composition and abundance changes

A longitudinal study by Koren et al. (21) using 16S rRNA gene sequencing analyzed the gut microbiota of pregnant women from the first trimester to the postpartum period. The results showed that the overall microbial diversity decreased during pregnancy, particularly in the third trimester. At the phylum level, there was an increase in the relative abundance of Proteobacteria and Actinobacteria, while the abundance of Firmicutes and Bacteroidetes, which are dominant in non-pregnant individuals, showed variable changes. For example, the ratio of Firmicutes to Bacteroidetes decreased in some pregnant women. At the genus level, specific genera such as Bifidobacterium and Lactobacillus, known for their probiotic properties, often showed a decline in abundance during pregnancy, while genera like Escherichia-Shigella increased (22).

These changes in the gut microbiota composition are influenced by multiple factors. Hormonal changes during pregnancy, such as increased levels of estrogen, progesterone, and human chorionic gonadotropin, can affect the gut environment. Progesterone, in particular, can slow down gastrointestinal motility, which may lead to changes in the gut microbiota by altering the transit time of food and the availability of nutrients for the microbes (23).

#### 3.1.2 Functional changes

The functional profile of the gut microbiota also changes during pregnancy. There is an increased abundance of microbial genes related to carbohydrate metabolism, lipid metabolism, and vitamin biosynthesis. For instance, genes involved in the metabolism of SCFAs, which are important metabolites produced by the gut microbiota, are upregulated. SCFAs, such as acetate, propionate, and butyrate, play crucial roles in energy metabolism, immune regulation, and maintenance of gut barrier function. The increased production of SCFAs during pregnancy may contribute to the energy needs of the mother and the developing fetus (24).

Moreover, the gut microbiota in pregnant women shows enhanced ability to metabolize bile acids. Bile acids are important for lipid digestion and absorption, and their metabolism by the gut microbiota can influence the enterohepatic circulation of bile acids, which is altered during pregnancy to meet the increased demand for lipid metabolism (25).

### 3.2 The role of gut microbiota in a healthy pregnancy outcome

A balanced gut microbiota is essential for a healthy pregnancy outcome, contributing to various aspects of maternal and fetal health through multiple mechanisms.

#### 3.2.1 Immune regulation

The gut microbiota plays a crucial role in training and modulating the maternal immune system during pregnancy. It helps to establish a state of immune tolerance towards the semi-allogeneic fetus, preventing excessive immune responses that could potentially lead to pregnancy complications such as RSA. The gut microbiota interacts with immune cells in the GALT, including dendritic cells, T cells, and B cells. For example, certain commensal bacteria can induce the differentiation of regulatory T cells (Tregs), which are key players in maintaining immune tolerance. A study by Dengke Qin et al. found that patients with RSA exhibit a notable enrichment of CD24+ DSCs, which can secrete 3-hydroxyisovaleric acid and impede the induction of naïve CD4+ T cells into Tregs (26). Tregs can suppress the activation of effector T cells, thus preventing an over-active immune response against the fetus (27). During pregnancy, a maternal high fiber diet can produce higher SCFAs and then promote Tregs differentiation through increased autoimmune regulator (Aire) expression in the thymus of offspring (28).

In addition, the gut microbiota can influence the production of cytokines, which are important signaling molecules in the immune system. A healthy gut microbiota promotes the production of anti-inflammatory cytokines such as interleukin-10 (IL-10) and transforming growth factor-β (TGF-β), while reducing the production of pro-inflammatory cytokines like tumor necrosis factor-α (TNF-α) and IL-6. This cytokine balance is essential for a successful pregnancy, as excessive inflammation has been associated with an increased risk of pregnancy loss (29, 30).

#### 3.2.2 Nutrient metabolism

The gut microbiota is involved in the digestion and absorption of nutrients, which is vital for the growth and development of the fetus. It can break down complex carbohydrates, proteins, and fats into simpler forms that can be absorbed by the host. For example, the fermentation of dietary fiber by the gut microbiota produces SCFAs, which are not only an energy source for the colonocytes but can also be absorbed into the bloodstream and utilized by other tissues. SCFAs can also influence lipid and glucose metabolism in the mother, helping to maintain normal metabolic homeostasis during pregnancy. A study by Ley et al. (31) demonstrated that germ-free mice, which lack a gut microbiota, had altered lipid metabolism and were more susceptible to diet-induced obesity compared to conventionally colonized mice.

Furthermore, the gut microbiota is involved in the synthesis of certain vitamins, such as vitamin K and B-vitamins. Vitamin K is essential for blood clotting, and its deficiency during pregnancy can increase the risk of bleeding complications. B-vitamins, including folate, are crucial for fetal neural tube development. The gut microbiota can contribute to the production and availability of these vitamins, ensuring proper fetal development (32).

#### 3.2.3 Maintenance of gut barrier function

The gut microbiota helps to maintain the integrity of the gut barrier, which is important for preventing the translocation of harmful bacteria and their toxins into the bloodstream. A healthy gut microbiota can stimulate the production of mucus by goblet cells in the intestinal epithelium, which forms a protective layer against pathogens. It can also enhance the expression of tight-junction proteins, such as occludin and zonula occludens-1 (ZO-1), which are responsible for sealing the gaps between intestinal epithelial cells. By maintaining the gut barrier function, the gut microbiota can prevent systemic inflammation and infection, which are risk factors for pregnancy complications (33).

In summary, the normal changes in the gut microbiota during pregnancy are part of an adaptive process to support the physiological needs of the mother and the developing fetus. Healthy gut microbiota is essential for a successful pregnancy outcome, contributing to immune regulation, nutrient metabolism, and maintenance of gut barrier function. Any disruption to this delicate balance, leading to gut microbiota dysbiosis, may potentially increase the risk of pregnancy-related complications, including RSA.

## 4 Dysbiosis of gut microbiota and RSA

### 4.1 Altered gut microbiota composition in RSA patients

The rapid development of DNA sequencing technology has greatly changed our understanding of microbial communities in various complex environments. In order to compare the gut microbiota of patients with RSA with that of normal pregnant women, fecal and other samples were collected from patients with RSA and healthy pregnant women. These samples were cultured and analyzed qualitatively to determine the differences between the two groups. In addition, 16S sequencing technology was also used. 16S sequencing is a high-throughput technology for analyzing bacterial community composition in a specific environment or habitat. It can study the diversity, abundance and community structure of microorganisms in environmental samples, and provide insights into the relationship between microorganisms and their host or environment. Numerous studies have demonstrated significant differences in the gut microbiota composition between RSA patients and healthy pregnant women, suggesting that gut microbiota dysbiosis may be associated with the occurrence of RSA.

Cui et al. uncovered that the bacterial abundance index decreased in RSA patients, but the bacterial diversity index increased. They also found that Roseburia significantly decreased while Ruminococcus significantly increased in RSA patients. Furthermore, in RSA patients with intrauterine adhesion, Klebsiella significantly increased, while Prevotella.9 and Roseburia significantly decreased (34). Yao et al. reported that Coprococcus3 and Odoribacter were linked to a reduced risk of RSA, while the Eubacterium ruminantium group was associated with an increased risk of RSA (35). A case-control study by Liu et al. (36) compared the gut microbiota of 41 women with RSA and 19 healthy pregnant controls using 16S rRNA gene sequencing. The results showed that the *α*-diversity, including the Chao1 estimators and Shannon index, was significantly lower in the RSA group, indicating a reduced microbial diversity. The relative abundance of Bacteroidetes genera known as the dominant bacteria community in the gastroenteric environment of healthy humans was decreased, while the abundance of Firmicutes was increased in RSA patients. At the genus level, there was a decrease in beneficial bacteria such as Prevotella, Selenomonas, and Gammaproteobacteria in the RSA patients, while potentially pathogenic bacteria like Prevotellaceae, Bacteroidales, and Eubacterium ruminantium were more abundant. Another study by Jin et al. (37) also found similar results. They analyzed the gut microbiota of 20 RSA patients and 20 healthy controls. The RSA group had a higher relative abundance of Proteobacteria and a lower abundance of Bacteroidetes compared to the controls. In terms of specific genera, the levels of yeast, Enterococcus, and Enterobacter were significantly higher in RSA patients, while the levels of Lactobacillus and Bifidobacterium were lower, which were consistent with the findings of Liu et al. (36). With this observation, an overly diverse and imbalanced microbiome could be detrimental to pregnancy outcomes.

The possible reasons for these differences are multi-factorial. Firstly, dietary habits may play a role. Unhealthy diets, such as high-fat, high-sugar, and low-fiber diets, are more common in some RSA patients. These diets can promote the growth of certain pathogenic bacteria while inhibiting the growth of beneficial bacteria, leading to gut microbiota dysbiosis (38). For example, a diet rich in simple carbohydrates can increase the growth of Escherichia-Shigella, which may disrupt the normal balance of the gut microbiota. Secondly, stress is prevalent among RSA patients due to the psychological burden of repeated pregnancy losses. Chronic stress can affect the gut-brain axis, leading to changes in gut motility, mucus production, and immune function, all of which can influence the composition of the gut microbiota (39). Stress-induced release of hormones such as cortisol can alter the gut environment, making it more favorable for the growth of some harmful bacteria. Moreover, the use of medications, especially antibiotics, in RSA patients can also disrupt the gut microbiota. Antibiotics not only kill pathogenic bacteria but also destroy the normal commensal bacteria in the gut, leading to a decrease in microbial diversity and an imbalance in the gut microbiota composition (40). If a patient with RSA has a history of taking antibiotics for other infections, it may contribute to the observed gut microbiota dysbiosis (Table 2).

**Table 2:** Changes of microbial abundance and composition at phylum and genus/species/levels.

Taxonomic level	Bacterial taxon	Change (↑/↓)	Function relevance
Phylum	Proteobacteria	↑	Dominant in disturbed/nutrient-enriched environments; includes many pathogens
	Bacteroidetes	↑	Major carbohydrate degraders; common in healthy gut/soil
	Firmicutes	↓	Butyrate producers; affected by stress/dysbiosis
	Actinobacteria	↓	Organic matter decomposition; sensitive to environmental change
	Chloroflexi	↑	Anaerobic degradation; increases in anoxic conditions
Genus/species	*Lactobacillus* spp.	↓	Probiotics; decreased under pathogenic infection
	*Bacteroides fragilis*	↓	Anti-inflammatory; reduced in dysbiosis
	*Escherichia/Shigella*	↑	Opportunistic pathogens; bloom under stress
	*Faecalibacterium prausnitzii*	↓	Major butyrate producer; depleted in inflammation
	*Aeromonas veronii*	↑	Fish pathogen; significantly increased post-infection
	*Klebsiella*	↑	Carbohydrate fermenter, reduced in inflammatory bowel disease
	*Prevotella*	↓	Butyrate produces gut health
	*Roseburia*	↓	Fermentative; promotes gut health
	*Coprococcus*	↓	Cellulolytic; complex carbs
	*Odoribacter*	↓	Supporting gut barrier
	*Eubacterium ruminantium*	↑	Anaerobic; involved in polysaccharide breakdown
	*Selenomonas*	↓	Commensals
	*Gammaproteobacteria*	↓	Increased in gut dysbiosis
	*yeast*	↑	Enterovirus infections-
	*Enterococcus*	↑	Pathogenic; associated with infection
	*Enterobacter*	↑	hospital-acquired infections
	*Bifidobacterium*	↓	Probiotic; maintains gut homeostasis

Therefore, maternal can improve their gut microbiota by adjusting their diet (increasing dietary fiber, supplementing probiotics) and managing stress (such as mindfulness, exercise), etc. under the guidance of doctors to provide a better internal environment for the pregnancy.

### 4.2 Potential mechanisms linking gut microbiota dysbiosis to RSA

#### 4.2.1 Immune-mediated mechanisms

The gut microbiota plays a crucial role in modulating the immune system, and dysbiosis can disrupt this balance, leading to immune-mediated pregnancy complications such as RSA. One of the key aspects is the imbalance between T helper 17 (Th17) and Treg cells (41).

Th17 cells are a subset of CD4+ T cells that secrete pro-inflammatory cytokines, such as IL-17, IL-21, and IL-22. These cytokines can promote inflammation and are involved in the defense against extracellular pathogens. In contrast, Treg cells are regulatory T cells that secrete anti-inflammatory cytokines, such as IL-10 and TGF-β, and play a vital role in maintaining immune tolerance (42).

In a normal pregnancy, a proper balance between Th17 and Treg cells is essential for maintaining immune tolerance towards the fetus while still providing protection against infections. However, gut microbiota dysbiosis can disrupt this balance. Studies have shown that certain commensal bacteria in the gut can promote the differentiation of Treg cells. For example, Bifidobacterium and Lactobacillus can produce metabolites that stimulate the development of Treg cells, such as SCFAs (43). In RSA patients, the reduced abundance of these beneficial bacteria, as mentioned earlier, may lead to a decrease in Treg cell differentiation.

Conversely, the increased abundance of potentially pathogenic bacteria, like Escherichia-Shigella in RSA patients, can promote the differentiation of Th17 cells. These bacteria can activate pattern-recognition receptors on immune cells, such as Toll-like receptors (TLRs), triggering a cascade of signaling pathways that lead to the production of cytokines that favor Th17 cell differentiation (44). A recent study further demonstrated that in women with unexplained miscarriage, specific gut microbiota alterations are associated with increased serum levels of Th1/Th17 cytokines (including IL-17A, IL-17F, and TNF-α), and that microbe-associated metabolites are positively correlated with these cytokine changes, providing direct evidence for the microbiota-immune axis in pregnancy loss (36). The resulting imbalance, with an increased Th17/Treg ratio, can lead to a pro-inflammatory environment at the maternal-fetal interface. This pro-inflammatory state can damage the placental tissue, disrupt the normal placental-fetal circulation, and ultimately increase the risk of pregnancy loss (41).

In addition to Th17/Treg cell imbalance, gut microbiota dysbiosis can also affect the production of other cytokines. For instance, it can lead to an overproduction of pro-inflammatory cytokines such as TNF-α and IL-6. These cytokines can activate immune cells, including natural killer (NK) cells and macrophages, at the maternal-fetal interface. Excessive activation of these cells can cause damage to the trophoblast cells, which are essential for placental development and function. High levels of TNF-α can induce apoptosis of trophoblast cells, while IL-6 can disrupt the normal communication between the mother and the fetus, both of which can contribute to RSA (45).

#### 4.2.2 Metabolic disorders

The gut microbiota is involved in various metabolic processes, and its dysbiosis can lead to metabolic disorders that are associated with RSA. One of the most well-studied metabolites produced by the gut microbiota is SCFAs.

SCFAs, mainly acetate, propionate, and butyrate, are produced by the fermentation of dietary fiber by the gut microbiota. In normal pregnancy, SCFAs play important roles in energy metabolism, immune regulation, and maintenance of gut barrier function. However, in RSA patients, the production of SCFAs may be disrupted due to gut microbiota dysbiosis. A study by Li et al. (46) found that the fecal levels of SCFAs, especially butyrate, were significantly lower in women with RSA compared to healthy pregnant controls.

Butyrate, in particular, has been shown to have anti-inflammatory and immunomodulatory effects. It can act on immune cells, such as macrophages and T cells, to reduce the production of pro-inflammatory cytokines and promote the production of anti-inflammatory cytokines. In the context of pregnancy, butyrate can also affect the function of trophoblast cells. It can enhance the invasion and migration of trophoblast cells, which are crucial for proper placental implantation and development. The reduced levels of butyrate in RSA patients may lead to impaired trophoblast function, increased inflammation at the maternal-fetal interface, and ultimately an increased risk of pregnancy loss (47).

Importantly, the impact of low SCFA levels extends beyond immune dysregulation to directly influence endocrine pathways. Butyrate serves as a primary energy source for intestinal epithelial cells, and its deficiency can compromise gut barrier integrity, leading to systemic inflammation. More directly relevant to the gut-uterus axis, SCFAs can modulate the sensitivity of endometrial cells to steroid hormones. Emerging evidence suggests that gut microbiota-derived metabolites can influence steroid hormone metabolism through the regulation of hydroxysteroid dehydrogenases (HSDs), enzymes produced by bacterial phyla including Actinobacteria, Proteobacteria, and Firmicutes that are essential for steroid hormone activation and inactivation (48). This provides a direct mechanistic link whereby altered SCFA production due to dysbiosis could affect local progesterone metabolism and bioavailability at the maternal-fetal interface. Thus, low SCFA status represents a key metabolic node connecting dysbiosis to both local immune imbalance and potential endocrine dysfunction.

Another metabolite that has been implicated in the relationship between gut microbiota and RSA is trimethylamine N-oxide (TMAO). TMAO is produced by the oxidation of trimethylamine (TMA), which is derived from the metabolism of dietary nutrients, such as choline, carnitine, and phosphatidylcholine, by the gut microbiota. High levels of TMAO have been associated with various metabolic disorders, including cardiovascular disease and insulin resistance (49).

In the context of pregnancy, a study (50) found that TMAO directly activates nuclear factor-*κ*B (NF-*κ*B) and mitogen-activated protein kinase (MAPK) signaling pathways in endothelial cells, leading to increased production of pro-inflammatory cytokines (e.g., TNF-α, IL-6) and reactive oxygen species (ROS). A recent study on missed abortions further supports this connection, demonstrating through KEGG pathway enrichment analysis that gut microbiota dysbiosis is associated with significant alterations in lipid metabolism and inflammatory pathways, which may contribute to pregnancy loss (51). These mechanisms are directly relevant to the oxidative stress and inflammation observed at the maternal-fetal interface in RSA. It can also affect the function of endothelial cells in the placenta, leading to impaired blood flow and nutrient supply to the fetus. The increased oxidative stress and inflammation induced by high TMAO levels can disrupt the maternal-fetal interface and contribute to the occurrence of RSA (52). Therefore, alleviating inflammatory responses, enhancing antioxidant capacity, and enriching beneficial bacteria to maintain intestinal homeostasis are crucial for sustaining normal pregnancy (53).

#### 4.2.3 Endocrine disruption

The gut microbiota can also influence the reproductive endocrine system, and its dysbiosis may disrupt the normal hormonal balance, contributing to RSA.

Estrogen and progesterone are two key hormones in pregnancy. Estrogen is involved in the regulation of uterine growth, blood flow, and the development of the placenta, while progesterone is essential for maintaining pregnancy by suppressing uterine contractions and promoting immune tolerance (54).

The gut microbiota is increasingly recognized as a full-fledged endocrine organ that interacts with the reproductive endocrine system throughout a woman's lifetime by modulating estrogen, androgens, insulin, and other hormones (55). Sex steroids, derived mainly from gonads, can shape microbiota composition. A study by Silvia Diviccaro et al. investigated the relationship between sex, gut steroid production, and microbiota composition in rats (56). Using advanced analytical techniques, the researchers found that female rats had higher levels of certain steroids (pregnenolone, progesterone, and isoallopregnanolone) in their colon, while males had higher testosterone. Removing the gonads (gonadectomy) altered gut steroid production in a sex-dependent manner and significantly impacted microbiota composition. These findings reveal a novel sex-specific link between gut microbiota and local steroid production, with potential implications for the gut-brain axis. The gut microbiota can affect the metabolism of these hormones. For example, some bacteria in the gut, such as certain species of Bacteroides and Clostridium, can produce enzymes that are involved in the metabolism of estrogen. They can convert estrogens to their inactive forms or vice versa, thereby influencing the bioavailability of estrogen (57). In RSA patients, gut microbiota dysbiosis may lead to abnormal estrogen metabolism. The enzymatic machinery for steroid hormone metabolism is directly provided by gut bacteria. Specific bacterial phyla, including Actinobacteria, Proteobacteria, and Firmicutes, can produce HSDs that catalyze the interconversion of steroid hormones between their active and inactive forms (48). This bacterial-derived enzymatic activity represents a fundamental mechanism by which the gut microbiota directly regulates the local and systemic bioavailability of progesterone and other reproductive hormones. Dysbiosis leading to alterations in the abundance of HSD-producing bacteria could therefore directly trigger progesterone imbalance at the maternal-fetal interface. A study by Zhu et al. (6) found that the levels of some estrogen-metabolizing enzymes in the gut microbiota were altered in RSA patients, which may result in either reduced or excessive estrogen levels. Abnormal estrogen levels can disrupt the normal uterine environment, affect the growth and development of the placenta, and increase the risk of pregnancy loss (58).

Similarly, the gut microbiota can also impact progesterone metabolism. Although the exact mechanisms are not fully understood, it is hypothesized that the gut microbiota may influence the synthesis, transport, or degradation of progesterone. Progesterone deficiency has long been associated with an increased risk of miscarriage, as it is crucial for maintaining the quiescence of the uterus and supporting the early stages of pregnancy. Gut microbiota dysbiosis-induced alterations in progesterone metabolism may contribute to the development of RSA by disrupting the normal progesterone-mediated physiological processes (59).

Moreover, the gut microbiota-endocrine connection may also involve other hormones, such as insulin and thyroid hormones. Insulin resistance, which can be influenced by gut microbiota dysbiosis, is associated with an increased risk of pregnancy complications, including RSA. Abnormal thyroid function, which can also be affected by the gut microbiota, is another risk factor for pregnancy loss. The gut microbiota may interact with the hypothalamic-pituitary-thyroid axis, leading to changes in thyroid hormone levels and metabolism, which can impact pregnancy outcomes (60).

**Figure 1:**
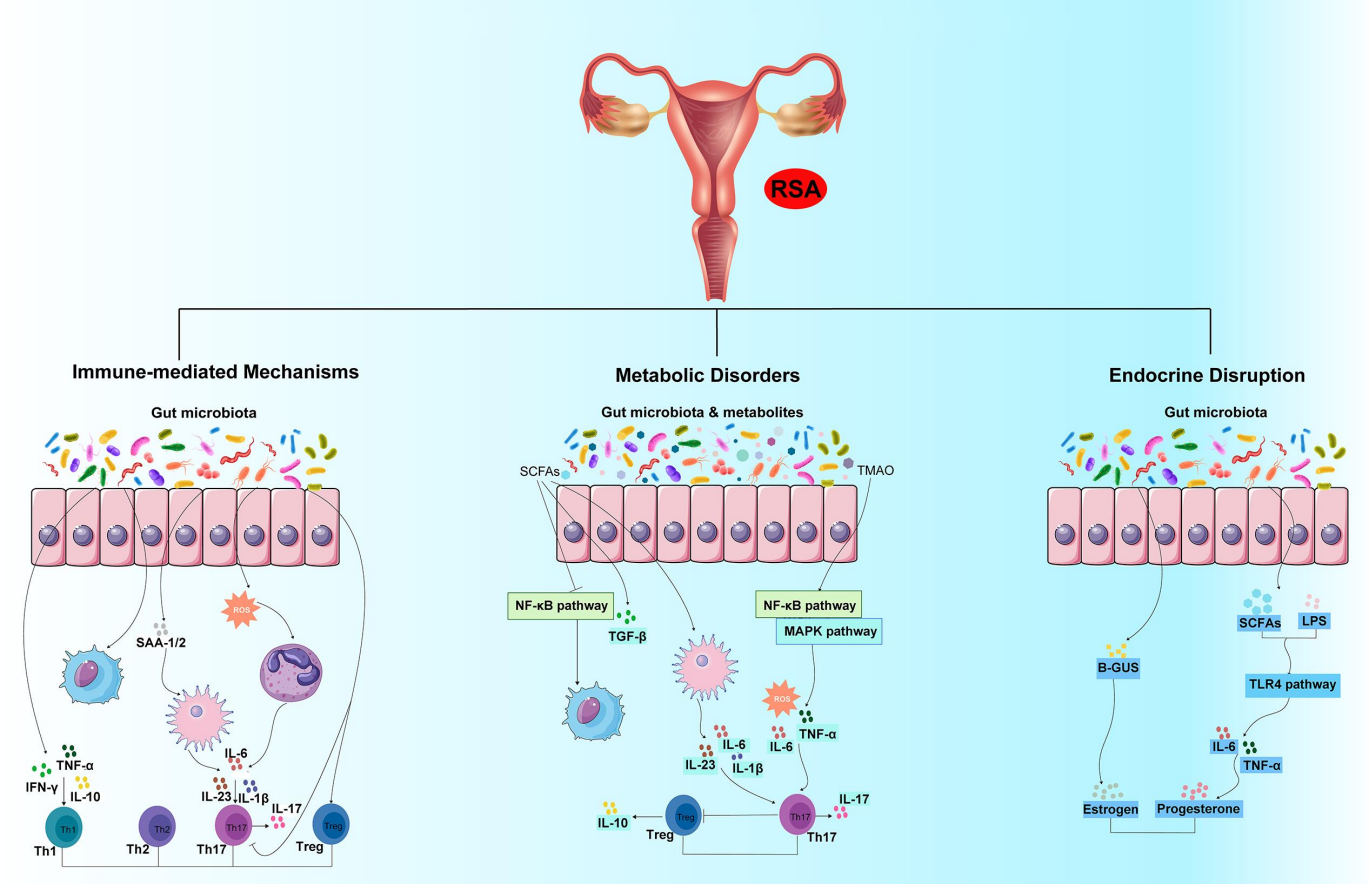
Potential mechanisms linking gut microbiota dysbiosis to spontaneous abortion (RSA). This figure illustrates the three primary pathways through which gut microbiota dysbiosis is proposed to contribute to the pathogenesis of RSA: immune-mediated mechanisms, metabolic disorders, and endocrine disruption. (1) Immune-mediated mechanisms: Dysbiosis disrupts the delicate balance between pro-inflammatory and regulatory immune cells. An overabundance of pathogenic bacteria can promote the abnormal release of pro-inflammatory cytokines (such as TNF-α, IFN-*γ*, IL-23, and IL-6) and then regulate the differentiation of Th1, Th2, Th17 and Treg cells. (2) Metabolic disorders: Dysbiosis alters the production of key microbial metabolites, including short-chain fatty acids (SCFAs) and trimethylamine N-oxide (TMAO). Reduced levels of SCFAs can weaken their protective effects, lead to impaired Treg cells differentiation and increased the inflammation signaling pathway (such as NF-*κ*B pathway), linking metabolism to immune dysfunction. Elevated TMAO levels activate pro-inflammatory signaling pathways (NF-*κ*B and MAPK) in endothelial and immune cells, thus increasing the production of reactive oxygen species (ROS) and inflammatory cytokines, contributing to vascular dysfunction at the maternal-fetal interface. (3) Endocrine disruption: Dysbiosis can interfere with the metabolism and bioavailability of critical reproductive hormones. Alterations in the gut microbiota can affect the metabolism of estrogen and progesterone.

In summary, gut microbiota dysbiosis in RSA patients can lead to immune-mediated, metabolic, and endocrine disruptions, all of which can contribute to the increased risk of pregnancy loss (Figure 1). Understanding these mechanisms is crucial for developing new diagnostic and therapeutic strategies for RSA. However, these evidences limit direct mechanistic evidence from animal models or *in vitro* systems, and further studies should be deeply explored the potential mechanisms linking gut microbiota dysbiosis to RSA.

### 4.3 Causal relationship between gut microbiota dysbiosis and RSA

Available evidence supports a bidirectional causal relationship rather than a unidirectional link between gut microbiota dysbiosis and RSA (61). On one hand, dysbiotic gut microbiota can independently trigger immune imbalance, metabolic disorders, and endocrine disruption, thereby directly increasing the risk of miscarriage; evidence from fecal microbiota transplantation studies further validifies that abnormal gut microbiota alone is sufficient to induce adverse pregnancy outcomes (36, 46). On the other hand, repeated pregnancy loss causes sustained psychological stress, hormonal fluctuations, dietary alterations, and clinical interventions such as antibiotic use, all of which secondarily disrupt gut microbial structure and function, meaning dysbiosis may also arise as a consequence of RSA (62). Major limitations of current research include the lack of animal models that strictly conform to the clinical definition of RSA and insufficient mechanistic evidence to confirm causal links between specific bacterial strains, key metabolites (e.g., SCFAs), and RSA pathogenesis. Future longitudinal cohort studies and standardized fecal microbiota transplantation experiments are warranted to clarify the exact causal direction.

### 4.4 Heterogeneity and contradictory viewpoints in gut microbiota-RSA association

Despite overall consistent trends, considerable heterogeneity and contradictory findings exist across studies (63). Microbiota ***α*-diversity** shows inconsistent changes in RSA patients, with reports of decreased, increased, or unchanged diversity, likely due to variations in ethnicity, sample size, and sequencing regions (36). The **Firmicutes/Bacteroidetes ratio** and abundance of *Lactobacillus* and *Bifidobacterium* also lack consistent patterns across cohorts (12, 48). Similarly, the reported correlations of SCFAs and TMAO with RSA are not uniformly replicated, partly owing to differences in dietary habits and detection methodologies (49). Furthermore, racial and regional variations in gut microbiota composition lead to inconsistent RSA-associated microbial markers among populations worldwide (64). Collectively, these observations indicate that the association between gut microbiota and RSA is modulated by multiple confounding factors, and universal microbial biomarkers for RSA require further validation in large-sample, multi-center studies.

## 5 Clinical studies on gut microbiota and RSA

### 5.1 Observational studies (cohort studies)

Several cohort studies have been conducted to explore the relationship between gut microbiota and RSA, aiming to provide insights into the natural history of this association.

A prospective cohort study by Liu et al. (36) followed 60 women of reproductive age for one year. The participants’ gut microbiota was analyzed at baseline using 16S rRNA gene sequencing, and their pregnancy outcomes were tracked. The results showed that women with a lower richness and evenness in their gut microbiota had a significantly increased risk of RSA. And the diversity of gut microbiota is significantly lower with different microbiota profile in the miscarriage patients than the controls. Another cohort study by Jin et al. (37) focused on women with a history of RSA. The study enrolled 20 women with a history of at least two consecutive miscarriages and 20 healthy controls. The gut microbiota was analyzed before the next pregnancy attempt. The researchers found that the diversity of the gut microbiota was lower in the RSA group compared to the controls. At the genus level, the abundance of Escherichia-Shigella was significantly higher in the RSA group, while the abundance of beneficial bacteria such as Bifidobacterium and Lactobacillus was lower. Further research should focus on the differences in diet, genetic background or environmental factors among different racial groups, so as to make the results more reliable.

In summary, cohort studies have generally suggested an association between gut microbiota dysbiosis and an increased risk of RSA, with consistent findings regarding the altered abundance of certain bacterial phyla and genera, such as increased Proteobacteria and decreased Bifidobacterium and Lactobacillus in RSA patients. However, the results can vary depending on factors such as study population, sample size, and study design, indicating the need for larger-scale, multi-center cohort studies to further clarify this relationship.

### 5.2 Intervention studies (RCTs)

RCTs have been carried out to investigate the effects of interventions on the gut microbiota and pregnancy outcomes in women with a history of RSA.

One double-blinded randomized controlled trial enrolled 50 women with a record of at least three RIFs and 50 women with successful pregnancies as a control group (65). They found that probiotic intervention led to a reduction in Th1 cells and an increase in Th2 cells in RIF patients, as well as pro-inflammatory cytokine expression decreased, and anti-inflammatory cytokine expression increased. In addition, a case-control study explored the association between dietary habits and risk of spontaneous abortion (66). The results showed that the risk of spontaneous abortion was inversely and significantly related to green vegetables, fruit, milk, cheese, eggs and fish consumption. These changes in the gut microbiota and metabolites were associated with improved immune function, as evidenced by a more balanced Th1/Th2 cytokine profile at the maternal-fetal interface.

In conclusion, RCTs on interventions to modulate the gut microbiota in RSA patients have shown some promising results, with probiotic and dietary interventions potentially improving pregnancy outcomes by altering the gut microbiota composition and function. However, these studies are conducted based on small sample sizes and possess significant heterogeneity, which weakens the conclusiveness of the findings. More well-designed RCTs with large sample size are needed to optimize the choice of interventions, including the type of probiotics, the composition of the diet, and the timing and duration of treatment.

### 5.3 Systemic reviews and meta-analyses

Systematic reviews and meta-analyses have synthesized the available evidence on the relationship between gut microbiota and RSA, providing a more comprehensive and objective assessment of this association.

A recent systematic review and meta-analysis by Zhao et al. (67) included 15 studies (8 case-control studies and 7 cohort studies) that investigated the gut microbiota in RSA patients. The results showed that compared with healthy controls, RSA patients had a significantly lower relative abundance of Bacteroidetes [standard mean difference (SMD)= −0.75, 95% CI: −1.2 to −0.3, *P* < 0.01] and a higher relative abundance of Proteobacteria (SMD = 0.8, 95% CI: 0.35–1.25, *P* < 0.01). At the genus level, the relative abundance of Bifidobacterium (SMD = - 0.82, 95% CI: −1.3 to −0.34, *P* < 0.01) and Lactobacillus (SMD = −0.68, 95% CI: −1.1 −0.26, *P* < 0.01) was lower in RSA patients, while the relative abundance of Escherichia-Shigella (SMD = 0.9, 95% CI: 0.45–1.35, *P* < 0.01) was higher. The meta-analysis also found that the alpha—diversity of the gut microbiota was significantly lower in RSA patients (SMD = - 0.9, 95% CI: - 1.4–0.4, *P* < 0.01), indicating a reduced microbial diversity.

Another meta-analysis by Zhang et al. (68) focused on the effect of probiotic intervention on pregnancy outcomes in women with RSA. The analysis included 6 RCTs with a total of 500 participants. The results showed that probiotic supplementation significantly increased the live-birth rate [risk ratio (RR) = 1.5, 95% CI: 1.1–2.0, *P* = 0.01] and decreased the miscarriage rate (RR = 0.5, 95% CI: 0.3–0.8, *P* = 0.002) compared to placebo. The subgroup analysis showed that the beneficial effects of probiotics were more pronounced when the probiotic formulation contained multiple strains and was administered for at least three months before pregnancy.

However, the evidence from these systemic reviews and meta-analyses also has limitations. The included studies had heterogeneity in terms of study design, sample size, population characteristics, and methods of gut microbiota analysis. For example, different studies used different sequencing techniques and taxonomic classification methods to analyze the gut microbiota, which may have affected the comparability of the results. Additionally, some of the included RCTs had small sample sizes, which may have reduced the statistical power and the generalizability of the findings.

In summary, systemic reviews and meta-analyses have provided valuable evidence for the association between gut microbiota dysbiosis and RSA, as well as the potential beneficial effects of probiotic interventions. However, due to the existing heterogeneity and limitations in the primary studies, more high-quality research is needed to strengthen the evidence base and further clarify the relationship between gut microbiota and RSA.

Cohort Studies, RCTs, systematic reviews and meta-analyses have provided new insights into the association between the gut microbiota and RSA (Table 3). However, the existing studies may have some limitations. For instance, differences in the population, confounding factors, etc. can affect the reliability of the results. Therefore, in the future, a large sample and high-quality literature need to be included for further research.

**Table 3:** Research about relationship between gut microbiota and RSA.

Clinical studies	Author	Year	Results	Reference
Observational studies	Liu et al.	2021	Women with a lower richness and evenness in their gut microbiota had a significantly increased risk of RSA. The diversity of gut microbiota is significantly lower with different microbiota profile in the miscarriage patients than the controls.	(37)
	Jin et al.	2019	The diversity of the gut microbiota was lower in the RSA group compared to the controls. At the genus level, the abundance of Escherichia-Shigella was significantly higher in the RSA group, while the abundance of beneficial bacteria such as Bifidobacterium and Lactobacillus was lower.	(38)
Intervention studies	Kamrani A et al.	2025	Probiotic intervention led to a reduction in Th1 cells and an increase in Th2 cells in RIF patients, as well as pro-inflammatory cytokine expression decreased, and anti-inflammatory cytokine expression increased.	(61)
	Di Cintio E et al.	2001	The risk of spontaneous abortion was inversely and significantly related to green vegetables, fruit, milk, cheese, eggs and fish consumption. These changes in the gut microbiota and metabolites were associated with improved immune function, as evidenced by a more balanced Th1/Th2 cytokine profile at the maternal-fetal interface.	(46)
Systemic reviews and meta-analyses	Zhao et al.	2021	Compared with healthy controls, RSA patients had a significantly lower relative abundance of Bacteroidetes (standard mean difference [SMD]= −0.75, 95% CI: −1.2 to −0.3, *P* < 0.01) and a higher relative abundance of Proteobacteria (SMD=0.8, 95% CI: 0.35–1.25, *P* < 0.01). At the genus level, the relative abundance of Bifidobacterium (SMD=−0.82, 95% CI: −1.3 to −0.34, *P* < 0.01) and Lactobacillus (SMD = −0.68, 95% CI:−1.1–0.26, *P* < 0.01) was lower in RSA patients, while the relative abundance of Escherichia - Shigella (SMD=0.9, 95% CI: 0.45–1.35, *P* < 0.01) was higher. The alpha −diversity of the gut microbiota was significantly lower in RSA patients (SMD = −0.9, 95% CI: −1.4–0.4, *P* < 0.01), indicating a reduced microbial diversity.	(62)
	Zhang et al.	2019	Probiotic supplementation significantly increased the live-birth rate (risk ratio [RR] = 1.5, 95% CI: 1.1–2.0, *P* = 0.01) and decreased the miscarriage rate (RR = 0.5, 95% CI: 0.3–0.8, *P* = 0.002) compared to placebo. The subgroup analysis showed that the beneficial effects of probiotics were more pronounced when the probiotic formulation contained multiple strains and was administered for at least three months before pregnancy.	(63)

## 6 Current challenges and future perspectives

### 6.1 Challenges in research

Despite the growing body of research on the relationship between gut microbiota and RSA, several challenges remain that need to be addressed to further advance our understanding in this field.

#### 6.1.1 Sample size and generalizability

Many of the current studies, especially observational studies and some RCTs, have relatively small sample sizes. Small sample sizes limit the statistical power of the studies, making it difficult to detect subtle but important associations between gut microbiota alterations and RSA. For example, some case-control studies with only 40–120 participants in each group may not be sufficient to accurately identify all the relevant changes in the gut microbiota composition and their relationship to RSA (36, 37). This also affects the generalizability of the findings. Results obtained from a small, homogeneous group of patients may not be applicable to the broader population of RSA patients, who may have diverse genetic, environmental, and lifestyle backgrounds.

#### 6.1.2 Research methodological heterogeneity

There is significant heterogeneity in the research methods used across different studies. Different studies may use various techniques for gut microbiota analysis, such as 16S rRNA gene sequencing, metagenomic sequencing, or targeted metabolite analysis. The choice of sequencing platform, primers used in 16S rRNA gene sequencing, and the bioinformatics pipelines for data analysis can all lead to differences in the results (69). For instance, different primer sets may amplify different regions of the 16S rRNA gene, potentially leading to biases in the detection of certain bacterial taxa. In addition, the definition of RSA and the classification of pregnancy outcomes can also vary between studies, making it challenging to compare and synthesize the results.

#### 6.1.3 Limitations in microbiota analysis technologies

Current microbiota analysis technologies have their limitations. The differences in 16S rRNA gene sequencing, metagenomic sequencing and DNA extraction methods seriously undermined the comparability of the study. 16S rRNA gene sequencing, although widely used, has several drawbacks. 16S rRNA gene sequencing has primer bias due to the different amplification regions (such as V4 region and V3-V4 region), and can only identify at the genus level, resulting in contradictory judgments regarding beneficial and harmful bacteria related to RSA. Metagenomic sequencing, which can provide more comprehensive genetic information about the microbiota, is more expensive and complex to analyze. Although metagenomic sequencing has high resolution, the differences in sequencing depth (3G vs. 15G) and analysis procedures (various assembly software and databases) have led to the fragmentation of the conclusions regarding the functional association of the microbial community. Moreover, both techniques may have biases in DNA extraction and amplification, which can affect the accuracy of the results (70). These differences make it difficult to unify the various research conclusions and hinder the application and transformation of microbiome analysis in RSA studies. Additionally, these techniques mainly focus on bacteria, while the gut microbiota also includes archaea, fungi, and viruses, whose roles in RSA are still relatively under-explored due to the limitations of current analytical methods.

#### 6.1.4 Determining causality

Establishing a causal relationship between gut microbiota dysbiosis and RSA is a major challenge. Most of the current evidence is based on observational studies, which can only show associations. Even in RCTs, it is difficult to prove that the observed changes in pregnancy outcomes are directly caused by the manipulation of the gut microbiota. There may be confounding factors, such as underlying genetic predispositions, dietary habits, and other environmental factors, that can influence both the gut microbiota and the risk of RSA. For example, a woman's genetic background may affect both her gut microbiota composition and her susceptibility to immune-mediated disorders that can lead to RSA (71). Separating the direct effects of the gut microbiota from these confounding factors requires more sophisticated study designs and statistical methods.

### 6.2 Future research directions

To overcome the current challenges and further explore the relationship between gut microbiota and RSA, future research can be directed towards the following areas.

#### 6.2.1 Multi-omics studies

Integrating multiple omics technologies, such as metagenomics, metabolomics, and proteomics, can provide a more comprehensive understanding of the gut microbiota-host interaction in the context of RSA. Metagenomics can reveal the genetic potential of the gut microbiota, metabolomics can analyze the small-molecule metabolites produced by the microbiota and the host, and proteomics can identify the proteins involved in the relevant biological processes. For example, by combining metagenomic and metabolomic data, researchers can identify specific bacterial species in the gut microbiota that are associated with the production of key metabolites, such as SCFAs or TMAO, which are related to RSA (72). This integrated approach can help to elucidate the complex molecular mechanisms underlying the relationship between gut microbiota and RSA.

#### 6.2.2 Personalized treatment approaches

Given the high degree of individual variability in the gut microbiota, developing personalized treatment strategies for RSA patients based on their unique gut microbiota profiles holds great promise. Future research can focus on identifying specific gut microbiota biomarkers that can predict the risk of RSA and the response to different interventions, such as probiotic treatment or dietary modifications. For example, if certain bacterial taxa or metabolite profiles are found to be strongly associated with a high risk of RSA, targeted probiotic formulations can be developed to specifically modulate these biomarkers in individual patients (73). This personalized approach may lead to more effective prevention and treatment of RSA.

#### 6.2.3 Animal model validation

Animal models can play a crucial role in validating the findings from human studies and further exploring the causal relationship between gut microbiota and RSA. Mouse models, in particular, can be used to manipulate the gut microbiota through fecal microbiota transplantation (FMT), antibiotic treatment, or specific dietary interventions and then observe the effects on pregnancy outcomes. For example, by transplanting the gut microbiota from RSA patients into germ-free mice and comparing their pregnancy outcomes with mice receiving gut microbiota from healthy controls, researchers can directly test the role of the gut microbiota in RSA (74). Animal models also allow for more in-depth mechanistic studies, such as the examination of immune cell responses and gene expression changes in the placenta and uterus, which are difficult to perform in human subjects.

#### 6.2.4 Clinical application and translation

Translating the current research findings into clinical practice is an important future direction. This includes the development of standardized protocols for gut microbiota analysis in clinical settings, the establishment of guidelines for probiotic use in RSA patients, and the integration of gut microbiota-related biomarkers into routine prenatal care. For example, healthcare providers could use simple, non-invasive methods to analyze the gut microbiota of women planning pregnancy or those with a history of RSA and then provide personalized advice on diet, probiotic supplementation, or other interventions based on the results (75). Also, the characteristics of the gut microbiota can be used to conduct diagnostic stratification for patients with RSA, providing a basis for subsequent precise intervention. This could potentially improve the management of RSA and enhance the chances of successful pregnancy outcomes.

In conclusion, while significant progress has been made in understanding the relationship between gut microbiota and RSA, there are still many challenges to overcome. Future research in these areas has the potential to not only deepen our understanding of the underlying mechanisms but also lead to the development of novel diagnostic and therapeutic strategies for RSA, ultimately improving the lives of affected women.

## 7 Conclusion

This review confirms that gut microbiota dysbiosis is strongly associated with RSA via immune, metabolic, and endocrine pathways. RSA patients display consistent microbial signatures: reduced diversity, depleted *Bacteroidetes*, *Lactobacillus*, and *Bifidobacterium*, and elevated *Proteobacteria* and *Escherichia-Shigella*. The relationship is bidirectional: dysbiosis contributes to RSA, and RSA-related stress further disrupts microbial balance. Heterogeneity exists across populations and methods. Probiotics and dietary interventions show clinical potential. Future research should focus on longitudinal cohorts, mechanistic studies, and standardized microbiota-targeted therapies.
